# Salmonellosis in Lagos, Nigeria: Incidence of *Plasmodium falciparum*-associated Co-infection, Patterns of Antimicrobial Resistance, and Emergence of Reduced Susceptibility to Fluoroquinolones

**Published:** 2007-09

**Authors:** Kabir O. Akinyemi, Babajide S. Bamiro, Akitoye O. Coker

**Affiliations:** 1 Department of Microbiology, Lagos State University, Ojo PMB 1087, Apapa, Lagos, Nigeria; 2 Microbiology Research Laboratory, Department of Obstetrics and Gynecology, College of Medicine, University of Lagos, PMB 12005, Lagos, Nigeria; 3 Department of Medical Microbiology and Parasitology, College of Medicine, University of Lagos, Idi-Araba, PMB 12003, Lagos, Nigeria

**Keywords:** Antimicrobial resistance, Drug resistance, Microbial, Fluoroquinolones, *Plasmodium*, *Salmonella*, Salmonellosis, Nigeria

## Abstract

The present study was undertaken to examine the status of antimicrobial resistance in *Salmonella*-associated diseases, by verifying possible emergence of reduced susceptibility to fluoroquinolones in *Salmonella* isolates and determining the incidence of *Plasmodium falciparum*-associated co-infection with *Salmonella* serotypes. Antimicrobial resistance in clinical isolates of *Salmonellae* was examined for a 12-month period. Four hundred and forty-one patients comprising two groups were recruited. Group A comprised 235 patients diagnosed by clinicians of having pyrexia, and group B included stool samples of 206 patients presenting with gastroenteritis. Samples were cultured and isolates identified, and drug susceptibility testing was performed using the standard methods. Of the 235 samples screened in group A, 42 *Salmonella* isolates and 107 *Plasmodium* spp. were identified. Of the 42 *Salmonella* isolates, 19 (45.2%) were *Salmonella* Typhi, 9 (21.4%) S. Enteritidis, and 7 (16.7%) each of S. Paratyphi and S. Arizonae. *Plasmodium* spp.-associated co-infection with *Salmonellae* was observed in 16 patients mostly in complicated typhoidal cases and S. Enteritidis-associated bacteraemia. Fiftty-three of the 206 stool samples from group B patients were confirmed positive for bacterial pathogens, made up of 35 *Salmonella* and 18 *Shigella* isolates. Of the *Salmonella* isolates, 18 (51.4%) were S. Enteritidis, 11 (31.4%) S. Arizonae, 4 (11.4%) S. Paratyphi, and 2 (5.7%) S. Typhi. There was no statistically significant difference (p<0.01) in antimicrobial resistance patterns exhibited among typhoidal *Salmonellae* isolated in 2000 and 2005. A similar trend in resistance was recorded for non-typhoidal *Salmonellae* (p<0.05). For the first time in Lagos, Nigeria, *Salmonella* isolates (10–18%) with reduced susceptibility to both ciprofloxacin and ofloxacin at MIC_50_ and MIC_90_ values of 0.015 and 0.03 μg/mL respectively were found. Despite this development, ciprofloxacin and ofloxacin remain the drug of choice for severe cases of salmonellosis, although caution should be exercised by clinicians in their pres-criptions such that fluoroquinolone antibiotic therapy is used only in laboratory-proven cases of typhoid fever and *Salmonella*-associated bacteraemia to preserve its efficacy.

## INTRODUCTION

*Salmonella*, a genus of Gram-negative rod-shaped bacteria of the family Enterobacteriaceae, causes a wide range of human diseases, such as enteric fever, gastroenteritis, endocarditis, and bacteraemia (1). *Salmonella*-associated infections do not present with distinct clinical features: other bacterial, viral and even protozoans may mimic its presentations (2). More than 2,300 *Salmonella* enterica serotypes have been described. Only S. enterica serovar Typhi, S. Paratyphi, S. Typhimurium, S. Enteritidis, S. Choleraesuis, S. Hadar, S. Virchow, and S. Dublin, among others, play important epidemiological and epizootiological roles (3). Although infections with non-typhoidal *Salmonellae* usually cause self-limi-ted diarrhoeal illness, serious sequelae, including meningitis, sepsis, and death, may occur, especially among infants and elderly persons (4).

Adequate sanitary measures have led to a decrease in cases of typhoidal *Salmonellae* in developed countries, such as the USA and the UK, where the incidence is low, and most cases are associated with travellers returning from endemic areas of developing countries (5). However, in these countries, nontyhoidal salmonellosis is common, and most of these cases are associated with outbreaks from contaminated meat, dairy products, and/or by crosscontamination from foods contaminated with *Salmonellae* (6,7). The situation is different in developing countries of Africa and southern Asia where typhoidal salmonellosis continues to pose a threat to public health with an estimated incidence of 33 million cases each year (8,9). In Nigeria, morbidity associated with illnesses due to *Salmonella* continues to be on the increase and, in some cases, resulting in death. Like other countries, treatment of patients has been based on the use of first-line antibiotics, such as chloramphenicol and co-trimoxazole, and the third-generation cephalosporins. However, efficacies of some of these drugs have been doubtful, following the emergence of multidrug resistance in *Salmonella* strains (10,11). Fluoroquinolones have been found to be efficacious both in vitro and in vivo in the treatment of severe *Salmonella*-associated illnesses, although strains with reduced susceptibility to ciprofloxacin among travellers have been reported in some parts of the globe (12). In malaria-endemic region, such as Nigeria, there exists an association between falciparum malaria and *Salmonella*-associated bacteraemia. Factors, such as improper sewage disposal, poor personal hygiene, poverty, and rapidly-increasing urbanization, have been attributed to frequent cases and deaths arising from these diseases (2,13–15).

Thus, high rate of hospitalization and prolonged illness of patients with *Salmonella*-associated diseases, as a result of treatment failure with empirical therapy, has been a great concern to public health. Active surveillance on salmonellosis in recent years is rare. Besides, there is a paucity of data on co-infection of *Salmonellae* with the malaria parasite in Nigeria. The present study was undertaken to examine the status of antimicrobial resistance in *Salmonella*-associated diseases, verifying possible emergence of reduced susceptibility to fluoroquinolones in *Salmonella* isolates and determining the incidence of *Plasmodium falciparum*-associated co-infection with *Salmonella* serotypes.

## MATERIALS AND METHODS

### Patient population, case definition, and sample collections

Four-hundred and forty-one patients, made up of group A and B were recruited for the study. They sought treatments at the following referral centres: Ikeja General Hospital, Lagos, Infectious Diseases Hospital Mainland, Lagos, Central Bank of Nigeria Clinics Satellite, Lagos, and Central Medical Laboratory Health Centre, Lagos, from October 2004 to September 2005. Important bio-data, history of vaccination, antimicrobial therapy, time of onset of illness, etc. of these patients were recorded. Group A comprised 235 patients diagnosed by clinicians of having pyrexia (temperature ≥38.5 ^o^C) for up to five days with or without one or combination of the following symptoms: vomiting, headache, diarrhoea, loss of appetite, abdominal pain, and nausea. Only blood samples were requested from the patients due to the early onset of symptoms. Four mL of blood was collected from each patient. Of this, 1 mL of blood was transferred into a sterile EDTA bottle and kept at 4°C until the malaria-parasite test was performed. The remaining 3 mL of blood was directly inoculated into 27 mL of brain heart infusion (BHI) broth (Oxoid, UK) for bacteriological culture. Group B comprised 206 patients who sought medical care due to passing of frequent stool (diarrhoea) for two or more days without fever. Fresh stool samples were collected from this category of patients in Cary-Blair tubes (10 mL/tube), and the tubes containing samples were transported to the laboratory for bacteriological culture.

### Bacteriology and malariology

Stool samples were cultured on MacConkey (MC) agar, desoxycholate citrate (DC) agar, *Salmonella*-*Shigella* (SS) agar, and Selenite F broth (Oxoid, UK). Media were incubated overnight at 37°C, and non-fermenting lactose colonies of different morphological types growing on SS agar, DC agar, or MC agar plates were identified based on their biochemical properties. The BHI broth-culture bottles were inoculated at 37°C aerobically for 18–24 hours. Subcultures were made onto solid media plates of SS agar, MC agar, and DC agar and were further incubated at 37°C aerobically for 18–24 hours. In negative cases, subcultures were repeated from the broth daily for seven consecutive days and were then discarded. Colonies were first subjected to biochemical tests as described by Cowan and Steel (16). The commercially-available identification system—API 20E (bio-Mérieux, France)—was used. Colonies considered to be of Salmonella spp. were further tested for somatic (O) and flagella (H) antigens with polyvalent antisera (Wellcome Diagnostic, UK). For each patient, a 1 mL of blood sample was screened using the thick-blood smear technique for the detection of malaria parasite (17). Blood smear was prepared and air-dried. Freshly-prepared Giemsa stain was added, left for 45 minutes, and then rinsed off under slow-running tap-water. The slide was observed under an oil immersion lens for evidence of *Plasmodium* spp.

### Antimicrobial susceptibility testing and determination of minimum inhibitory concentration

Susceptibility patterns of *Salmonella* serotypes to 13 antibiotics were determined by the agar-diffusion method (18). Three to five colonies were inoculated into a tube containing tryptic soy broth (Difco, USA) and incubated overnight at 37°C. Standardization of the inocula was performed by diluting the broth cultures until turbidity matched the 0.5 McFarland standards. A sterile cotton swab was dipped into the standardized suspension, drained, and used for inoculating 20 mL of Mueller-Hinton agar in a 100-mm disposable plate (Sterlin, UK). The inoculated plates were air-dried, and antibiotic discs (Oxoid, UK) were placed on the agar using flamed forceps and were gently pressed down to ensure contact. The discs with the following were used for susceptibility testing: ampicillin (25 μg), chloramphenicol (30 μg), co-trimoxazole (25 μg), tetracycline (50 μg), nalidixic acid (30 μg), ciprofloxacin (20 μg), ofloxacin (20 μg); colistin-sulphate (10 μg), streptomycin (25 μg), gentamicin (10 μg), cefotaxime (30 μg), cefoperazone (30 μg), and amoxicillin (30 μg). The plates were incubated aerobically at 37°C for 18–24 hours. The diameters of the zones of inhibition were measured with a ruler and compared with a zone-interpretation chart (18). *Escherichia coli* ATCC 25922 was used as control. The determination of MIC was performed using the reference broth microdilution method recommended by the National Committee for Clinical Laboratory Standards (19). The reagent powders were dissolved in accordance with the instructions of the manufacturer (Sigma, Deisenhofen, Germany), diluted with Mueller-Hinton broth, and distributed to the wells of microdilution trays. Each tray was inoculated with approximately 5×10^5^ CFU per well to yield a final volume of 0.l mL per well. The trays were incubated at 35°C for 18 hours. E. coli ATCC 25922 was used as control strain in each test batch. MIC was taken as the highest dilution of the antibiotic that yielded no visible bacterial growth (no turbidity) when compared with the control. Thereafter, MIC_50_ and MIC_90_ were evaluated accordingly (20).

## RESULTS

**Fig. 1:**
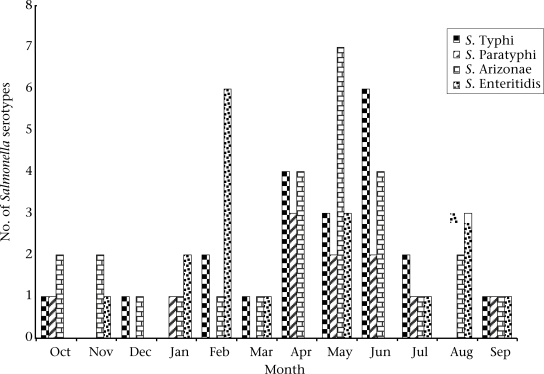
Monthly isolation of *Salmonella* strains from patients in Lagos, October 2004-September 2005

Of the 235 samples screened in group A patients, 42 *Salmonella* isolates and 107 *Plasmodium* spp. were identified. Of the 42 *Salmonella* isolates, S. Typhi was the most frequently-encountered serotype accounting for 45.2%, followed by S. Enteritidis (21.4%) (Table 1). Sixteen *Salmonella* spp. made up of seven each of S. Typhi and S. Enteritidis, and two of S. Paratyphi were isolated with *Plasmodium* spp. from patients with complications. These dual infections occurred in all age-groups studied, with predilection in the age-group of 16–30 years (Table 2). Fiftty-three of the 206 stool samples from group B patients were confirmed positive for bacterial pathogens, made up of 35 *Salmonella* and 18 *Shigella* isolates. Of the *Salmonella* isolates, S. Enteritidis (51.4%) was the most prevalent serotype. Other serotypes isolated were S. Arizonae (31.4%), S. Paratyphi (11.4%), and S. Typhi (5.7%) (Table 1). Salmonellae were isolated in each month from October 2004 to September 2005 with the peak incidence in May, June, and February. The least cases of *Salmonella*–associated illnesses occurred in March and December (Fig. 1). In this study, S. Typhi and S. Paratyphi were 100% resistant to ampicillin, approximately 90% to tetracycline, and 80% to chloramphenicol and gentamicin. The resistance of both the serotypes to cefotaxime, streptomycin, and co-trimoxazole ranged from 51.5% to 70%. Moreover, less than 20% reduced susceptibility to fluoroquinolones (ciprofloxacin and ofloxacin) was observed in both the serotypes. Similar antibiotic resistance patterns were observed and recorded for non-typhoidal *Salmonellae* isolated in the study (Table 3). Minimum inhibitory concentrations (MICs) of each of the 13 antibiotics that constituted 50% and 90% *Salmonellae* susceptible *in vitro* are shown in Table 4. For example, fluoroquinolones inhibited the growth of 50% and 90% of the isolates at 0.015 and 0.03 μg/mL respectively, whereas a wide variation in MIC_50_ and MIC_90_ values was recorded for other antibiotics, particularly ampicillin, chloramphenicol, and tetracycline, in the study.

**Table 1:** Absolute figures and percentages of pathogens isolated according to the source

Source (sample size)	No. of positive samples	*Salmonella enterica* serotypes isolated					Other pathogens obtained (% from total positive samples)	
		No. of isolates	S. Typhi	S. Paratyphi	S. Enteritidis	S. Arizonae	*Plasmodium* spp.	*Shigella* spp.
Blood (235)	152 (74.2)	42 (27.6)	19 (45.2)	7 (16.7)	9 (21.4)	7 (16.7)	107 (70.4)	3 (2.0)
Stool (206)	53 (25.8)	35 (66.0)	2 (5.7)	4 (11.4)	18 (51.4)	11 (31.4)	NA	18 (34.0)
Total	205 (100)	77 (100)	21 (27.3)	11 (14.3)	27 (35.1)	18 (23.4)	NA	21 (10.2)

Figures in parentheses indicate percentages; NA=Not applicable

**Table 2:** Relative risk of co-infection due to *Salmonellae* with *Plasmodiun* spp. in different age-groups of patients with pyrexia studied

	Patients with *Plasmodium* spp. (n=107)		Patients with *Salmonellae* (n=42)	
Age-groups (years)	Co-infection of patients with *Salmonellaspp* spp. (n=16)	Without *Salmonellaspp* spp. (n=91)	Co-infection of patients with *Plasmodium* spp. (n=16)	Without *Plasmodium* spp. (n=26)
0–5	2	27	2	1
6–15	3	28	3	7
16–30	5	17	5	10
31–45	4	11	4	4
≥46	2	8	2	4

**Table 3:** Comparison and percentage of resistant *Salmonella* serotypes to 13 antibiotics in two different studies from Lagos

Antibiotic used	Percentage of *Salmonella* serotypes in 2000∗ and the present study						
	*S.* Typhi		*S.* Paratyphi		*S.* Arizonae		*S.* Enteritidis†
	2000∗ (n=68)	Present study (n=21)	2000∗ (n=17)	Present study (n=11)	2000∗ (n=16)	Present study (n=18)	Present study (n=27)
Ampicillin	91.2	100	88.2	100	93.8	100	100
Co-trimoxazole	61.8	71.4	64.7	63.6	56.3	61.1	85.2
Chloramphenicol	73.5	85.7	70.6	81.8	87.5	88.9	85.2
Gentamicin	29.4	81.0	29.4	81.8	31.3	83.3	88.9
Tetracycline	64.7	90.5	64.7	90.1	68.8	88.9	92.6
Streptomycin	32.4	66.7	35.3	63.6	37.5	72.2	59.3
Cefotaxime	51.5	66.7	35.3	54.5	31.5	55.5	59.3
Nalidixic acid	13.2	38.1	17.7	27.3	12.5	27.8	33.3
Ciprofloxacin	0.0	14.3	0.0	18.2	0.0	11.1	14.8
Ofloxacin	0.0	14.3	0.0	18.2	0.0	11.1	14.8
Colinstin sulphate	44.1	52.4	41.2	27.3	37.5	55.6	40.7
Amoxicillin	ND	57.1	ND	63.6	ND	55.6	59.3
Cefoperazone	ND	57.1	ND	72.3	ND	61.1	55.6

∗ Data obtained from reference (10);

† Data not available; ND=Not determined; n=Number of isolates evaluated

**Table 4:** Minimun inhibitory concentrations (μg/mL) of 13 antibiotics each tested against *Salmonella* species isolated

Antibiotics used (break-point for resistance)	Typhoidal *Salmonellae* (n=32)			Non-typhoidal *Salmonellae* (n=45)		
	Range	MIC_50_	MIC_90_	Range	MIC_50_	MIC_90_
Ampicillin (>32)	64–128	64.0	128.0	64–128	64.0	64.0
Co-trimoxazole (>32)	0.25–64	16.0	64.0	0.25–64	16	64.0
Chloramphenicol (>16)	1–64	64.0	64.0	0.25–64	32	32
Gentamicin (>8)	4–64	1.0	32.0	0.25–16	16	16
Tetracycline (>8)	4–128	64.0	128.0	1–64	16	32
Streptomycin (>32)	1–128	64.0	128.0	1–128	64	64
Cefotaxime (>64)	32–64	64.0	64	16–64	64	64
Nalidixic acid (>8)	1–64	8.0	16.0	1–32	8.0	16
Ciprofloxacin (>4)	0.003–1	0.015	0.03	0.003–1	0.015	0.03
Ofloxacin (>8)	0.003–1	0.015	0.03	0.003–1	0.015	0.03
Colinstin sulphate (>8)	1–128	32.0	64.0	1–64	32	32
Amoxicillin (>32)	1–32	32.0	32.0	1–32	32	32
Cefoperazone (>64)	4–64	64.0	64.0	1–64	64	64

## DISCUSSION

Seventy-seven *Salmonella* strains, which included 42 (17.8%) from blood of patients with pyrexia and 35 (17.0%) from stool samples of patients with gastroenteritis, were isolated in the study, given an incidence of 17.5% of *Salmonella*-associated illnesses for the 12-month period. The results of the study indicated that the frequency of isolation of S. Typhi (45.2%) from patients with pyrexia has not changed from the previous report in Lagos in which 105 *Salmonella* isolates, made up of 67.3% S. Typhi, 16.8% S. Paratyphi, and 15.8% S. Arizonae, were obtained (10). The high prevalence was similar to non-typhoidal salmonellosis reported in Malawian and Tanzanian patients (21) and S. Typhi mostly implicated in India, South East Asia, and Latin America (9,12).

Despite a high endemic nature of malaria in Nigeria, information on *Salmonella* spp.-associated co-infection with *Plasmodium* spp. is scarce. We found in the blood of 16 patients *Plasmodium* spp.-associated co-infection with *Salmonella* isolates distributed among the age-groups examined. Seven of 16 each were S. Typhi and S. Enteritidis serotypes, while the rest were S. Paratyphi. The fact that these *Salmonella* serotypes were isolated from the blood of the pyrexia patients ruled out the possibilities that these individuals might be carriers. Previous workers elsewhere had documented an association between falciparum malaria and *Salmonella*-associated bacteraemia in malaria-endemic regions (15). Similarly, co-infections of bacteria in the family Enterobacteriaeciae with falciparum malaria have been reported in some malaria-endemic African countries (22,23). Our results were consonant with findings of these studies, thus representing the first published case in Lagos, Nigeria. Furthermore, we observed that nine of the 16 patients with dual infections suffered from serious typhoid and paratyphoid fever-related complications, such as delirium, confusion, coma, and spleenomegaly. Also, co-infection of *Plasmodium* spp. with S. Enteritidis-associated bacteraemia was observed in seven patients in this study. Generally, these observations could be associated with the fact that *Salmonella*-associated infection exacerbates infection due to P. *falciparum* and, thus, might account for complications in these patients. *P. falciparum* increases the risk of complications via a number of mechanisms, including immune dysfunctions (24). We also observed that 16 *Salmonella* isolates with *P. falciparum*-associated co-infection were resistant to eight or more antibiotics, including the third-generation cephalosporins. The implication of this is treatment failure and the probable emergence of multiple antimicrobial resistance in *Salmonella* strains.

There was no statistically significant difference (p<0.01) in the patterns of antimicrobial resistance in typhoidal *Salmonellae* isolated from cases of pyrexia in the present study and that of previous years (Table 4). However, the first decreased susceptibility to fluoroquinolones in *Salmonella* isolates at MIC_50_ and MIC_90_ values of 0.015 and 0.03 μg/mL respectively in Nigeria was recorded in this study. This observation is a concern as the only hope of therapy for typhoid fever and complicated cases of non-typhoidal salmonellosis in our environment is now threatened. Additionally, more than 51% of the isolates were resistant to cefotaxime and cephoperazone. This trend is of particular concern because the extended spectrum cephalosporins are the antibiotics of choice for children (25). In African countries, such as Kenya, Malawi, and Ethiopia, neither fluoroquinolones nor third-generation cephalosporin resistance among *Salmonella* strains is a problem (26).

In Nigeria, the observed reduced susceptibility to fluoroquinolones is a new scourge, and the explanation for this is inconclusive and difficult. This is because these drugs are relatively expensive as the cost of ciprofloxacin or ofloxacin is over $5.00 for a five-day therapy in adults. However, reports elsewhere have indicated that the use of antimicrobials for growth-promotion prophylaxis and treatment of food-animals increases the prevalence of resistance in human pathogens, particularly non-typhoidal salmonellosis. This practice is common in Asia and the United Kingdom and in some other European countries where fluoroquinolones have been approved for animal use (27,28). The situation is rare in our environment. However, the increasing smuggling of poultry products into our country, as a result of the ban in importation of these products in recent times, might be, among other factors, a likely reason for the emergence of reduced susceptibility to fluroquinolones in our environment. There has been a suggestion that the use of antimicrobials in animal growth should be phased out gradually, since similar benefits could be obtained by improving other aspects of animal care, such as hygiene (29). Denmark voluntarily suspended the use of all animal growth promoters in 1999 and has started yielding positive effect, and Switzerland also did the same in 2000 (30). It is saddening to say that, while these countries have made tremendous efforts in solving the problem of fluoroquinolone resistance in *Salmonella* serotypes, our environment has just witnessed the emergence of decreased susceptibility to fluoroquinolones in *Salmonella* isolates. The fear is that, if the trend continues, it may cause a serious public-health threat, thus, putting the problem into international pers-pective.

In conclusion, *Plasmodium* spp.-associated co-infection with *Salmonella* serotypes was observed more in patients with typhoidal complications and *Salmonella*-associated bacteraemia. We record the first reduced susceptibility of fluoroquinolones in *Salmonella* isolates in our environment. Despite this development, ciprofloxacin and ofloxacin remain the drug of choice for typhoidal and severe cases of non-typhoidal salmonellosis, although caution should be exercised by clinicians in their prescription such that fuoroquinolone antibiotic therapy is used only in laboratory-proven cases to preserve its efficacy. More importantly, there is a need for the Government to enforce the existing laws that prohibit smuggling of all kinds and the sale of drugs by unauthorized people. On a global scale, the current efforts of the World Health Organization towards establishing more National Reference Laboratories for Infectious Diseases and Antimicrobial Surveillance Centres in many countries should be intensified, and such efforts should benefit developing countries. This may pave the way for adequate records, proper monitoring, and effective management of antimicrobial resistance in a pathogen currently threatening the healthcare system.
